# Oxidative damage-induced hyperactive ribosome biogenesis participates in tumorigenesis of offspring by cross-interacting with the Wnt and TGF-β1 pathways in IVF embryos

**DOI:** 10.1038/s12276-021-00700-0

**Published:** 2021-11-30

**Authors:** Yue Huang, Zhiling Li, En Lin, Pei He, Gaizhen Ru

**Affiliations:** 1 Department of Reproductive Center, The First Affiliated Hospital of Shantou University Medical College, Shantou University, 515000 Shantou, Guangdong China; 2 Institute of Molecular Physiology, Shenzhen Bay Laboratory, 518000 Shenzhen, Guangdong China

**Keywords:** Embryology, Cancer

## Abstract

In vitro fertilization (IVF) increases the risk of tumorigenesis in offspring. The increased oxidative damage during IVF may be involved in tumor formation. However, the molecular mechanisms underlying this phenomenon remain largely unclear. Using a well-established model of oxidatively damaged IVF mouse embryos, we applied the iTRAQ method to identify proteins differentially expressed between control and oxidatively damaged zygotes and explored the possible tumorigenic mechanisms, especially with regard to the effects of oxidative damage on ribosome biogenesis closely related to tumorigenesis. The iTRAQ results revealed that ribosomal proteins were upregulated by oxidative stress through the Nucleolin/β-Catenin/n-Myc pathway, which stimulated ribosomes to synthesize an abundance of repair proteins to correct the damaged DNA/chromosomes in IVF-derived embryos. However, the increased percentages of γH2AX-positive cells and apoptotic cells in the blastocyst suggested that DNA repair was insufficient, resulting in aberrant ribosome biogenesis. Overexpression of ribosomal proteins, particularly Rpl15, which gradually increased from the 1-cell to 8-cell stages, indicated persistent hyperactivation of ribosome biogenesis, which promoted tumorigenesis in offspring derived from oxidatively damaged IVF embryos by selectively enhancing the translation of β-Catenin and TGF-β1. The antioxidant epigallocatechin-3-gallate (EGCG) was added to the in vitro culture medium to protect embryos from oxidative damage, and the expression of ribosome-/tumor-related proteins returned to normal after EGCG treatment. This study suggests that regulation of ribosome biogenesis by EGCG may be a means of preventing tumor formation in human IVF-derived offspring, providing a scientific basis for optimizing in vitro culture conditions and improving human-assisted reproductive technology.

## IVF: Mechanism and correction of increased cancer risk

Increased production of ribosomes, the multi-molecular structures inside cells where protein synthesis occurs, is implicated in the enhanced risk of cancer in offspring conceived by in vitro fertilization (IVF), suggesting an approach to reduce this risk. In studies on IVF mouse embryos, Zhiling Li and colleagues at First Affiliated Hospital of Shantou University Medical College in China found that harmful effects of natural oxidizing agents induced overproduction of specific protein components of ribosomes. This increased the embryos’ susceptibility to tumorigenesis through a particular molecular signaling pathway. Exposure to the natural antioxidant chemical epigallocatechin-3-gallate (EGCG) corrected the overproduction and protected the embryos. EGCG, found in green tea, is being widely investigated for its anticancer effects. Exposing embryos to EGCG during IVF might protect the resulting children from increased risk of tumor formation in later life.

## Introduction

In vitro fertilization (IVF) involves the manipulation of early embryos at a time when they may be particularly vulnerable to external disturbances. Environmental influences during embryonic development in vitro affect an individual′s susceptibility to epigenetic alterations and diseases such as cardiovascular disease, raising concerns about the potential consequences of IVF on the long-term health of offspring. The influence of IVF on tumor formation in offspring has been a topic of great concern for many researchers. There is increasing evidence that children who are born after IVF treatment have an increased risk of cancer^1,2^. A large registry-based study found a 42% higher risk of cancer in children conceived through IVF^3^. A 10-year follow-up study demonstrated that the total incidence of neoplasms was higher among IVF-conceived children (1.5/1000 person-years) than among naturally conceived children (0.59/1000 person-years; *P* < 0.001), and the association between IVF and total pediatric neoplasms remained significant after controlling for confounders (HR 2.48, 95% CI 1.71–3.50)^4^. However, the molecular mechanisms underlying this phenomenon remain largely unknown.

In vitro culture conditions, such as the culture medium, temperature, light, and oxygen concentration, which are unable to fully simulate the in vivo development environment, can lead to oxidative stress due to the production of excessive reactive oxygen species (ROS), causing developmental arrest in IVF-derived embryos. We have been researching the impacts of oxidative stress on IVF-derived embryos. In our previous studies, we treated mouse zygotes with different doses of hydrogen peroxide (H_2_O_2_) and found that 0.03 mM H_2_O_2_ was the minimum effective concentration able to reduce the blastocyst formation rate (without reducing the rates of 2-, 4-, and 8-cell embryo formation) by inducing DNA damage and chromosome aneuploidy, producing a model that strongly resembles the clinical phenomenon of oxidative damage in embryos during IVF^5–7^. DNA damage and chromosome aneuploidy are closely associated with tumorigenesis^8,9^. Cells from precancerous lesions and even hyperplastic lesions accumulate DNA alterations, confirming that the gradual accumulation of DNA damage leads to cancerous transformation^8^. Oxidative damage may be one of the most important reasons why IVF-conceived children have an increased risk of pediatric neoplasms. To date, there is a lack of animal experimental research to reveal the effects and molecular mechanisms of oxidative damage on tumorigenesis in IVF-derived offspring. Therefore, we used an H_2_O_2_-induced model of oxidatively damaged IVF mouse embryos to study the tumorigenic mechanisms. It is also the first time that animal experiments have been applied to elucidate the possible pathogenesis of tumors in offspring derived from oxidatively damaged IVF embryos. Oxidative damage participates in tumorigenesis of offspring, possibly by altering the ribosome function of IVF-derived embryos.

Eukaryotic cytoplasmic ribosomes are large ribonucleoprotein complexes made up of small 40S and large 60S subunits. The 40S subunit consists of 18S ribosomal RNA (rRNA) and 33 different ribosomal proteins (Rps), whereas the 60S subunit consists of 25S, 5.8S, and 5S rRNA together with 47 ribosomal proteins (Rpl). Ribosome biogenesis involves the production and correct assembly of four rRNAs and 80 ribosomal proteins. Ribosomes are the molecular machines that produce all cellular proteins during a process called translation^10^. Precise regulation of ribosome biogenesis is fundamental for maintaining normal cell growth and proliferation^11^. Increasing evidence has underscored that multifaceted relations link abnormal ribosome biogenesis to cancer^12^. Ribosome biogenesis is abnormally regulated in tumors under strong growth pressure and is mainly upregulated to increase the rate of protein synthesis^13^. More importantly, most ribosomal proteins can influence proliferation and metastasis by performing extraribosomal regulatory functions involving binding to select critical target mRNAs^14^. Hyperactive ribosome biogenesis can promote tumorigenesis via extraribosomal functions, including regulation of oncogene activation and tumor suppressor gene silencing and contribution to the activation of tumor-related signaling pathways^15^. According to the literature, ribosomal proteins can be chemically modified by ROS, which may alter ribosome functions^16^. However, no reports have been published on the alterations in ribosome functions induced by oxidative damage in IVF-derived embryos or on the role of abnormal ribosome function in tumorigenesis in offspring.

In this study, using a well-established model of oxidatively damaged IVF mouse embryos, we aimed to explore the relevant mechanisms of tumorigenesis in IVF-derived offspring, especially the effects of ROS on ribosome biogenesis closely related to tumorigenesis. In addition, the antioxidant (-)-epigallocatechin gallate (EGCG) has been shown to exert not just a promoting effect on embryo development but also a cancer chemopreventive effect by modulating various cell signaling pathways, such as inducing apoptosis, reducing proliferation, and regulating angiogenesis^17,18^. Therefore, our further experiments focused on whether EGCG can antagonize ROS-induced alterations in ribosome biogenesis, which may be a means of preventing tumor formation in human offspring from IVF-derived embryos.

## Materials and methods

### Animals

Adult Kun-Ming mice (male: 3–6 months old; female: 4–8 weeks old) were obtained from the animal center of Shantou University Medical College. All work was carried out in accordance with the International Guiding Principles for Biomedical Research Involving Animals (2012 version) issued by the Council for the International Organizations of Medical Sciences. All experimental protocols were approved by the Laboratory Animal Ethics Committee of Shantou University Medical College (SUMC2014–014).

### Reagents

Detailed information on the materials used in our experiment is listed in Supplementary Table 1.

### Sperm and oocyte collection, in vitro fertilization, and embryo culture

As described in our previous study^7^, sperm harvested from the murine caudae epididymis were moved to capacitation medium (human tubal fluid medium containing 1.5% bovine serum albumin) and incubated in an incubator (37 °C, 5% CO_2_) for 1 h. Female mice were superovulated by sequential injection of 10 IU of pregnant mare serum gonadotropin and 10 IU of human chorionic gonadotropin 48 h apart. At 13–15 h after human chorionic gonadotropin administration, cumulus-oocyte complexes were obtained from the ovaries and placed in microdrops of fertilization medium (human tubal fluid medium supplemented with 0.4% bovine serum albumin) under paraffin oil. Capacitated sperm were added to each microdrop of fertilization medium containing oocytes and incubated under the conditions of 5% CO_2_ and 37 °C for 6 h. After fertilization, zygotes were transferred into embryo culture medium (human tubal fluid medium with both 0.4% bovine serum albumin and 10% fetal bovine serum) under oil in a 5% CO_2_ and 37 °C incubator.

### Mouse zygote model for oxidative damage

According to our previous study^5–7^, zygotes at 7 h postinsemination (hpi) were incubated in embryo culture medium containing 0.03 mM H_2_O_2_ in a 5% CO_2_ incubator at 37 °C for 30 min to obtain a model of oxidative damage in mouse zygotes.

### Addition of EGCG to IVF mouse embryos

Zygotes (6 hpi) were exposed to embryo culture medium containing different concentrations of EGCG (5, 10, 15, 20, 25, 30 μg/mL) on the basis of prior references and preliminary experiments^17^. Zygotes (7 hpi) were treated with 0.03 mM H_2_O_2_. After 30 min, zygotes were cultured in medium containing the corresponding concentrations of EGCG at 37 °C and 5% CO_2_. The dose effect of EGCG on blastocyst formation was investigated, and 20 μg/mL was considered the minimum effective concentration for improving the blastocyst formation rate of oxidatively damaged zygotes to the normal level.

### Determination of ROS products

Intracellular ROS levels were detected by the fluorogenic probe DCFH‐DA. A stock solution of DCFH‐DA (1 × 10^−3^ mol/L in dimethyl sulfoxide) was added to the embryo culture medium to a final concentration of 10 μmol/L. As described in an earlier report^7^, green fluorescence was observed under a fluorescence microscope (Nikon Eclipse 90 Ni-E) (Nikon, Tokyo, Japan), and Image-Pro Plus 6.0 (Media Cybernetics, Bethesda, MD, USA) was used to quantify the fluorescence.

### Immunofluorescence analysis

The protocol used for the immunofluorescence staining of embryos was previously described by our research group^7^. Zygotes were digested with 0.1% pancreatin for 30 s to remove zonae pellucidae, fixed in 4% paraformaldehyde for 30 min, and mounted on a polylysine-coated slide. Then, zygotes were permeabilized with 0.5% Triton X-100 in phosphate-buffered saline for 30 min, blocked with a sealing fluid for 1 h, and immunolabeled with primary antibody at 4 °C overnight, followed by Alexa Fluor 488 secondary antibody at room temperature for 1 h. Coverslips were mounted with ProLong Gold antifade mounting medium containing DAPI. Cells were visualized using a fluorescence microscope. The antibodies were optimized as described in Supplementary Table 1.

### TUNEL assay

A TUNEL assay was performed to analyze blastocyst apoptosis using the RiboAPO^TM^ One-Step TUNEL Apoptosis Detection Kit (red) in accordance with the manufacturer’s instructions^7^.

### Karyotype analysis

Zygotes were treated with hypotonic solution (0.9% sodium citrate in distilled water containing 3% distilled water) at 37 °C for 40 min and then transferred into fixative I (methanol: acetic acid: H_2_O = 5: 1: 2.5) for 5 min. When the color of the cell changed from brown to white and became subtransparent, the cell and a small amount of fixative II (methanol: acetic acid = 3: 1) were aspirated and released into a slide and immediately covered by a gentle flow of fixative II. The slide was placed into a Coplin jar filled with fixative II at room temperature overnight and then dipped into fixative III (methanol: acetic acid: H_2_O = 3: 3: 1) for 1 min. The chromosomes were stained with Giemsa for 30 min after air drying^7^. To avoid confusion, only one embryo burst after exposure to the fixative solutions, and the embryos were slowly mounted individually onto clean microscope slides. The slides were then scanned using a standard bright-field microscope (Nikon Eclipse 90 Ni-E).

### Isobaric tags for relative and absolute quantification (iTRAQ) analysis

#### iTRAQ labeling

Five hundred zygotes for each group (control and H_2_O_2_-treated groups) were prepared to extract protein. The protein concentration of the supernatant was determined by the Bradford method. The tryptic peptides were labeled with iTRAQ reagents (Applied Biosystems, Waltham, MA, USA) in accordance with the manufacturer’s instructions. The control and H_2_O_2_-treated groups were labeled with iTRAQ reagents 118 and 119, respectively.

#### LC–MS/MS analysis

The labeled samples were mixed together in equal amounts, and 200 μg of the mixture was then fractionated with a high pH RP-HPLC column. The sample was loaded onto the column in buffer A (98% ddH_2_O and 2% acetonitrile, pH 10) and eluted with the following gradient: an initial increase to 5% buffer B (98% acetonitrile and 2% ddH_2_O, pH 10), a subsequent 64 min linear gradient from 5 to 8% buffer B and successive ramps to 18%, 32%, and 95% buffer B, with a flow rate of 0.7 mL/min. The sample was analyzed by a Q-Exactive mass spectrometer (Thermo Fisher Scientific, Bremen, Germany), resuspended in solution C (99.9% ddH_2_O and 0.1% formic acid) and eluted with a gradient starting with 4% solvent D (99.9% acetonitrile and 0.1% formic acid) and ending at 95% solvent D with a flow rate of 350 nL/min for 70 min.

#### Database search

The MS/MS spectra were searched against the UniProt protein database of mice using Proteome Discoverer software (version 1.3; Thermo Fisher Scientific). The proteins with a fold change (the ratio of intensity of protein expression in H_2_O_2_-treated zygotes to control zygotes, 119/118) >1.5 were considered significantly differentially expressed proteins^7^. Gene Ontology (GO) and Kyoto Encyclopedia of Genes and Genomes (KEGG) were used to analyze the functional and pathway enrichment of differential genes.

### Western blot analysis

Lysates of 300 zygotes (the control and H_2_O_2_-treated groups) were obtained using RIPA lysis buffer containing protease and phosphatase inhibitors. Protein samples were separated by SDS–PAGE, transferred onto PVDF membranes, and probed with primary antibody at 4 °C overnight, followed by HRP-conjugated secondary antibody at room temperature for 1 h. Protein bands were visualized by a chemiluminescence kit. Optical densities of bands were determined with BandScan 5.0 software (Glyko, Novato, CA, USA)^7^. The antibodies were optimized as described in Supplementary Table 1.

### Real-time quantitative PCR analysis

Approximately 150 zygotes were prepared, and total RNA was extracted with an RNAprep pure Micro Kit. RNA samples were reverse-transcribed into cDNA using FastKing gDNA Dispelling RT SuperMix. Real-time quantitative PCR (RT–qPCR) analysis was performed by Talent qPCR PreMix (SYBR Green) on the CFX Connect^TM^ Real-Time PCR Detection System (BIO-RAD, CA, USA). The primers were described in Supplementary Table 2. RT–qPCR was conducted as described previously^19^. Relative mRNA expression normalized to GAPDH was calculated using the 2−ΔΔCT method.

### Statistical analysis

Data were collected from at least three independent experiments. Data expressed as the means ± standard deviations (SDs) were compared with Student’s *t* test. Data shown as percentages were analyzed using Pearson’s *χ*^2^ test. *P* < 0.05 was considered statistically significant. Statistical analysis was performed using SPSS software 19.0 (IBM, Armonk, NY, USA).

## Results

### Oxidative stress-induced developmental arrest, DNA damage, and chromosome aneuploidy in IVF mouse embryos

#### Oxidative stress

**Fig. 1:**
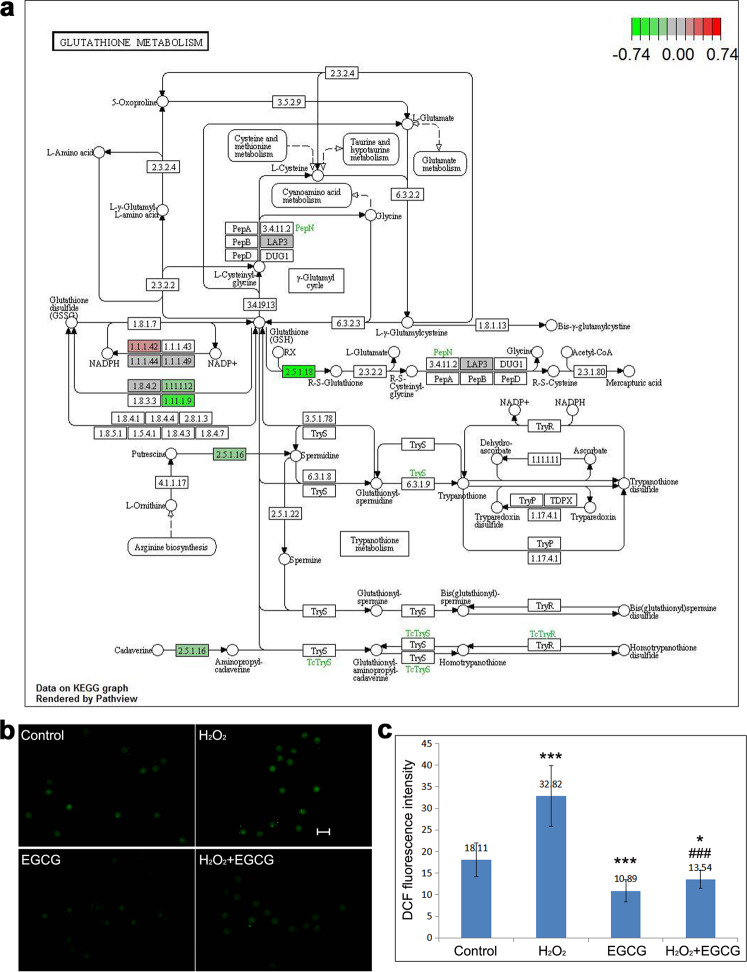
Oxidative stress in IVF embryos. **a** KEGG pathway map. KEGG analysis indicated that the downregulated proteins were mainly involved in glutathione metabolism, contributing to ROS accumulation and oxidative stress. **b** Representative fluorescence micrographs of ROS (green) in zygotes. Scale bar, 100 μm. **c** Average fluorescence intensity of ROS in zygotes. Data are expressed as the means ± SDs, collected from three independent experiments, and each experiment had at least five embryos. Differences among groups were analyzed using Student’s *t* test. *P < 0.05 and ***P < 0.001 vs. control, ^###^*P* < 0.001 vs. H_2_O_2_ treatment.

The iTRAQ technique can qualitatively and quantitatively analyze the dynamic changes of proteins in cells under different conditions, producing truly comprehensive information on cell function and process mechanisms^20^. In addition, due to its advantage of high sensitivity, the iTRAQ technique is more suitable than other techniques for gaining insights into the changes in proteomic profiles from samples comprising a very small number of cells, such as IVF-derived embryos. Therefore, we used iTRAQ labeling to identify proteins differentially expressed between the control and H_2_O_2_-treated zygotes and successfully found 93 upregulated and 147 downregulated proteins among 1464 total identified proteins, providing a powerful platform for us to study the possible mechanisms of tumorigenesis in IVF-derived offspring. GO and KEGG analyses indicated that H_2_O_2_ treatment resulted in decreased activity of glutathione peroxidase (Gpx4 and Gpx6) and glutathione transferase (Gstm1, Gstm5, Gstm7, and Gsto2), contributing to the accumulation of ROS in IVF-derived embryos (Fig. 1a).

The yields of ROS between different groups were compared using DCF fluorescence intensity in zygotes (7.5 hpi) (Fig. 1b, c). The mean DCF fluorescence intensity in zygotes of the H_2_O_2_-treated group was approximately twofold higher than that in zygotes of the control group (*P* < 0.001), which indicated that the production of ROS was augmented. EGCG (20 μg/mL) treatment significantly reduced ROS generation in the H_2_O_2_-treated zygotes to a level nearly three-quarters that of the control group (*P* < 0.001).

### Developmental arrest

**Fig. 2:**
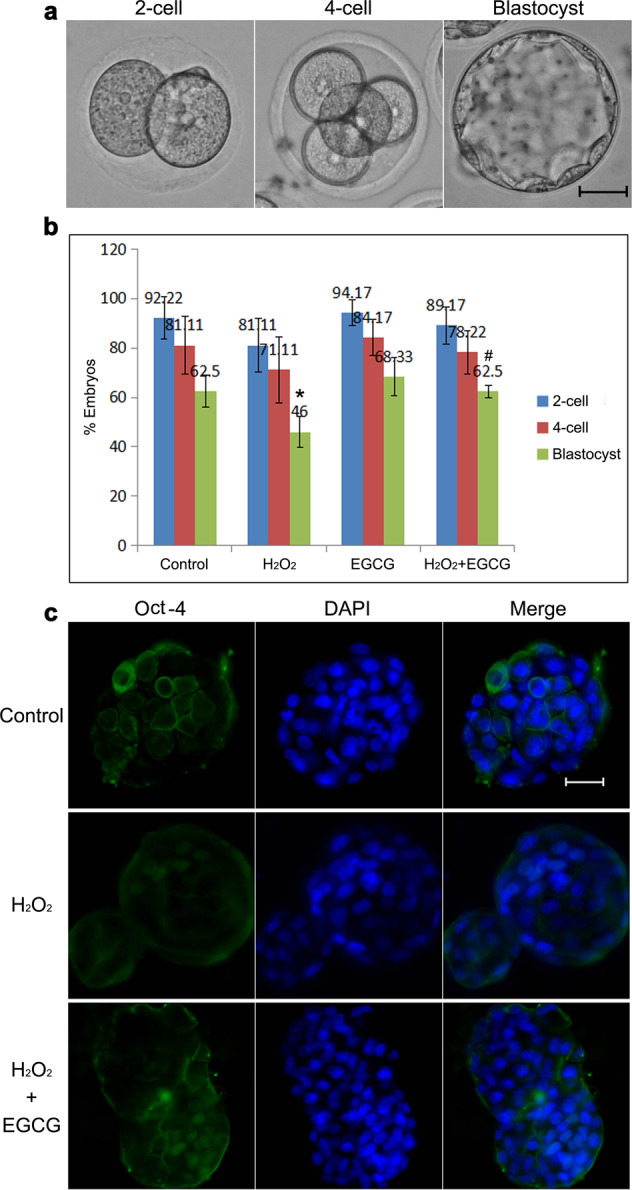
Developmental arrest in IVF embryos. **a** Representative images of 2-cell embryos, 4-cell embryos, and blastocysts. Scale bar, 20 μm. **b** Rates of 2-cell embryo, 4-cell embryo, and blastocyst formation. Data are expressed as the means ± SDs, collected from three independent experiments, and each experiment had at least five embryos. Differences among groups were analyzed using Student’s *t* test. *P < 0.05 vs. control, ^#^*P* < 0.05 vs. H_2_O_2_ treatment. **c** Representative fluorescence micrographs of ICM in blastocysts. The ICM cell number was determined by immunofluorescence staining for OCT-4 (green). DAPI staining (blue) was used to analyze the total cell number. Scale bar, 20 μm.

The cleavage rate and blastocyst formation rate were monitored to explore the effects of ROS and EGCG on embryo development in vitro (Fig. 2a, b). Compared to the control group, exposure of zygotes to 0.03 mM H_2_O_2_ did not clearly reduce the rates of 2- and 4-cell embryo formation (*P* > 0.05) but led to a significant reduction in the blastocyst formation rate (*P* < 0.05), which was similar to the findings observed clinically. Conversely, the addition of EGCG to H_2_O_2_-treated zygotes effectively promoted blastocyst formation, causing blastocyst formation to return to the normal level (*P* < 0.05 vs. H_2_O_2_ treatment). In addition, the developmental potential of IVF-derived blastocysts with respect to the total cell number and allocation to inner cell mass (ICM) and trophectoderm was evaluated (Fig. 2c and Table 1). The average number of ICM cells and the ratio of ICM to total cells in blastocysts in the H_2_O_2_-treated group were decreased to ~50% of the control values (*P* < 0.05), suggesting that oxidative stress could lead to embryo developmental arrest. In the blastocysts that developed from H_2_O_2_/EGCG-treated zygotes, EGCG treatment not only increased the ICM cell number, which was not significantly different from that of the control group (*P* < 0.05 vs. H_2_O_2_ treatment), but also significantly increased the blastocyst total cell number, which was nearly 1.5-fold higher than that of the control group (*P* < 0.05 vs. both H_2_O_2_ treatment and control). These results indicate that EGCG improves the development and quality of in vitro embryos.

**Table 1:** Comparison of ICM cell number and ICM/total cell ratio in IVF embryos with different treatments.

	ICM cell number	Total cell number	ICM/total cell ratio
Control group	14.33 ± 1.15	47.33 ± 4.51	30.06 ± 5.07
H_2_O_2_-treated group	7 ± 1^a^	41 ± 3	17.19 ± 3.12^a^
EGCG-treated group	17.9 ± 1.3	70.51 ± 3.5^a^	25.39 ± 1.75
H_2_O_2_ + EGCG-treated group	17.33 ± 2.08^b^	69.33 ± 5.51^a,b^	24.95 ± 1.36^b^

^a^*P* < 0.05 vs. control, ^b^*P* < 0.05 vs. H_2_O_2_ treatment.

#### DNA damage

**Fig. 3:**
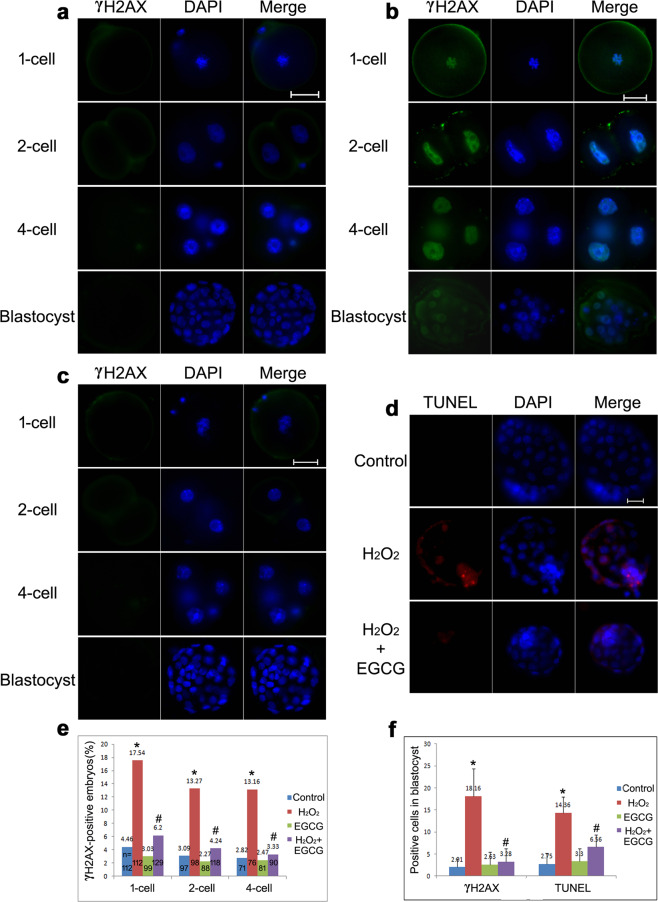
DNA damage in IVF embryos. **a**–**c** Representative fluorescence micrographs of γH2AX (green) in the control, H_2_O_2_-treated, and H_2_O_2_/EGCG-treated groups. **d** Representative fluorescence micrographs of TUNEL-positive apoptotic cells (red) in blastocysts. Nuclei are stained with DAPI (blue). Scale bar, 20 μm. **e** Rates of γH2AX-positive embryos at the 1-cell, 2-cell, and 4-cell stages. Data are shown as percentages collected from at least three independent experiments, and *n* shows the total number of embryos. Differences among groups were analyzed using Pearson’s *χ*^2^ test. *P < 0.05 vs. control, ^#^*P* < 0.05 vs. H_2_O_2_ treatment. **f** Rates of γH2AX- and TUNEL-positive cells in blastocysts. Data are expressed as the means ± SDs, collected from three independent experiments, and each experiment had at least five embryos. Differences among groups were analyzed using Student’s *t* test. *P < 0.05 vs. control, ^#^*P* < 0.05 vs. H_2_O_2_ treatment.

DNA damage was determined by measuring phosphorylated H2AX (γH2AX) using immunofluorescence staining, and apoptosis was detected by TUNEL assay (Fig. 3a–f). While there were few γH2AX foci and little TUNEL-positive staining in the control group, H_2_O_2_ treatment led to a remarkable increase in the proportion of γH2AX-positive embryos at the 1-cell, 2-cell, and 4-cell stages as well as in the percentages of γH2AX-positive cells and TUNEL-positive apoptotic cells in blastocysts (*P* < 0.05). These findings indicated that ROS caused DNA damage in IVF-derived embryos that was repaired by activation of the G2/M cell cycle checkpoint^6^, but the associated DNA repair was incomplete until the blastocyst stage, and thus, the transferred blastocysts appeared to consist of oxidatively damaged and apoptotic cells. As expected, no γH2AX foci or TUNEL staining was observed in the majority of H_2_O_2_/EGCG-treated embryos (*P* < 0.05 vs. H_2_O_2_ treatment). EGCG protected embryos from ROS-induced DNA damage and increased the undamaged cells in preimplantation blastocysts.

#### Chromosomal aneuploidy

**Fig. 4:**
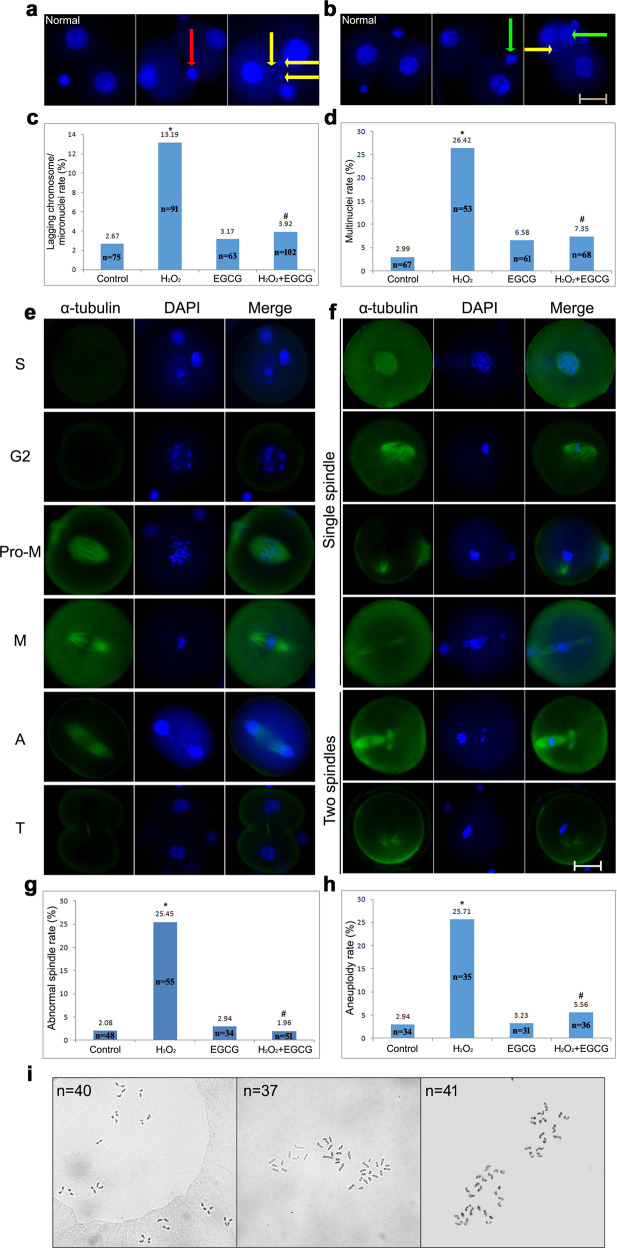
Chromosome aneuploidy in IVF embryos. **a**, **b** Representative images of lagging chromosomes/micronuclei and multinuclei, respectively. The red arrow represents lagging chromosomes, the yellow arrow represents micronuclei, and the green arrow represents multinuclei. Scale bar = 20 µm. **c**, **d** Rates of lagging chromosomes/micronuclei and multinuclei, respectively. Data are shown as percentages collected from at least three independent experiments, and *n* shows the total number of embryos. Differences among groups were analyzed using Pearson’s *χ*^2^ test. *P < 0.05 vs. control, ^#^*P* < 0.05 vs. H_2_O_2_ treatment. **e**, **f** Representative fluorescence micrographs of normal and abnormal spindle formation, respectively. Cells were stained with anti-α-tubulin antibody (green) to detect microtubules, namely, the spindle. Pro-M, M, A, and T refer to prometaphase, metaphase, anaphase, and telophase, respectively. Scale bar = 20 μm. **g**, **h** Rates of abnormal spindles and aneuploidy, respectively. Data are shown as percentages collected from at least three independent experiments, and *n* shows the total number of embryos. Differences among groups were analyzed using Pearson’s *χ*^2^ test. *P < 0.05 vs. control, ^#^*P* < 0.05 vs. H_2_O_2_ treatment. **i** Representative images of chromosome karyotype.

We used DAPI staining to observe lagging chromosomes/micronuclei and multinuclei of IVF-derived embryos caused by chromosome mis-segregation during first mitosis (Fig. 4a–d). The rates of lagging chromosomes/micronuclei and multinuclei were found to increase with H_2_O_2_ treatment (*P* < 0.05). However, the rates in the H_2_O_2_/EGCG-treated group were significantly lower than those in the H_2_O_2_-treated group (*P* < 0.05) and were close to those in the control group (*P* > 0.05).

Historically, α-tubulin, the main component of spindle microtubules, has been believed to govern spindle microtubule dynamics during chromosome segregation^21^. We next examined the localization of α‐tubulin to assess abnormal spindle formation, which is closely associated with chromosome mis-segregation (Fig. 4e–g). In normal zygotes, newly nucleated microtubules self-organized into bipolar spindles during prometaphase; metaphase nuclei were predominantly condensed into tight bars aligned on the metaphase plate with two tight triangular bioriented microtubule arrays; chromosomes arrived at the cell poles, and spindle microtubules disappeared during telophase. In contrast, 25.45% (14/55) of the H_2_O_2_-treated zygotes exhibited features of aberrant microtubule formation, including loss of spindle bipolarity, microtubule breakage, monopolar spindles, and elongated spindles accompanied by chromosome misalignment at metaphase, which may act as a trigger for micronuclei formation (vs. control 2.08% [1/48], *P* = 0.002). It is worth mentioning that two bipolar spindles were observed in a few H_2_O_2_-treated metaphase zygotes; however, the two spindles failed to align and come into close apposition to form a compound barrel-shaped structure, producing 2-cell embryos with one or two binucleated blastomeres, which provides a potential rationale for the multinucleated blastomere formation observed in human IVF-derived embryos^22^. In contrast, the H_2_O_2_/EGCG-treated zygotes presented no obvious spindle abnormalities, and only one cell with chromosome breakage was observed (1.96 [1/51] vs. H_2_O_2_ treatment, *P* = 0.001).

Micronuclei and multinuclei are considered biomarkers of aneuploidy^23^, so we examined the karyotypes of 2-cell embryos to identify the effects of ROS and EGCG on aneuploidy (Fig. 4h, i). The aneuploidy rate in the control group was lower than that in the H_2_O_2_‐treated group (*P* < 0.05), revealing that ROS produced by the in vitro culture conditions may be one of the most important reasons for the high incidence of aneuploidy in IVF-derived embryos. This effect of ROS on aneuploidy was reversed by EGCG supplementation (*P* < 0.05), indicating that EGCG protects embryos in vitro from ROS-induced chromosome aneuploidy.

### Identification of differentially expressed proteins in response to oxidative damage by iTRAQ labeling

**Fig. 5:**
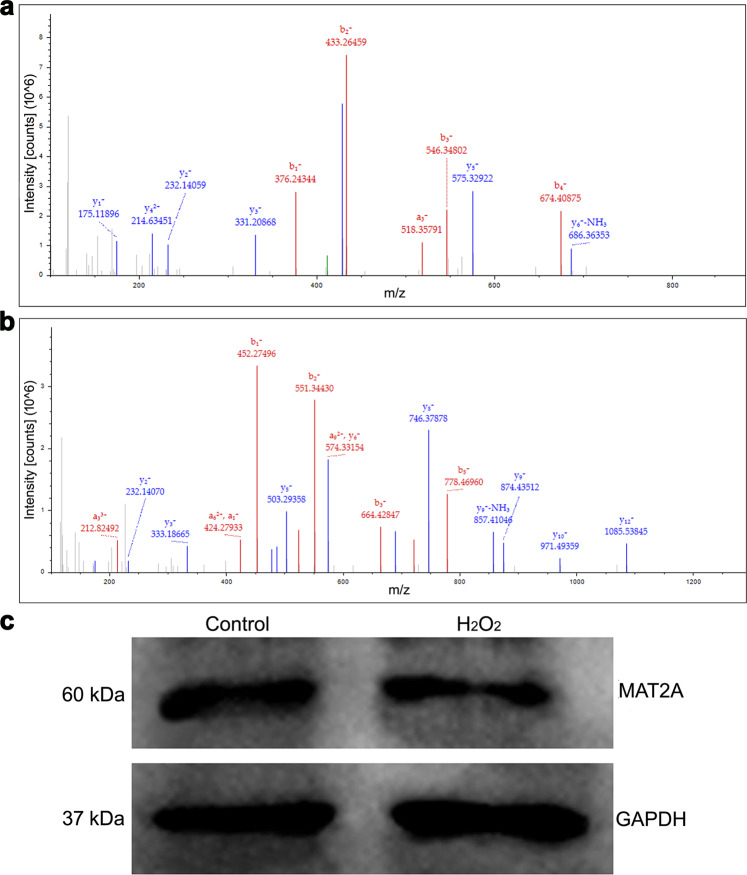
Identification of differentially expressed proteins in response to oxidative damage by iTRAQ. **a**, **b** MS/MS spectra used for the identification of H2A and MAT2A, respectively. **c** MAT2A protein levels were detected by western blotting to validate the iTRAQ results. GAPDH was used as a normalization control. Molecular weights are indicated on the left: MAT2 (60 kDa) and GAPDH (37 kDa).

DNA damage and aneuploidy formation induced by ROS during IVF may contribute to tumorigenesis in offspring. However, the molecular mechanisms underlying this phenomenon remain largely unclear. To determine possible mechanisms, we used iTRAQ labeling to identify proteins that were differentially expressed between the control and H_2_O_2_-treated zygotes. The MS/MS spectra used for the identification of H2A and Mat2a are shown in Fig. 5a, b. To validate the data obtained from the iTRAQ method, Mat2a protein levels were measured by western blotting and found to be suppressed by ROS in IVF-derived embryos (*P* < 0.05), consistent with the iTRAQ findings (Fig. 5c). All of the identified and differentially expressed proteins were analyzed according to the GO database and classified into “cellular component”, “molecular function”, and “biological process” subcategories (a given gene product may exhibit one or more functional annotations).

#### Cellular component

**Fig. 6:**
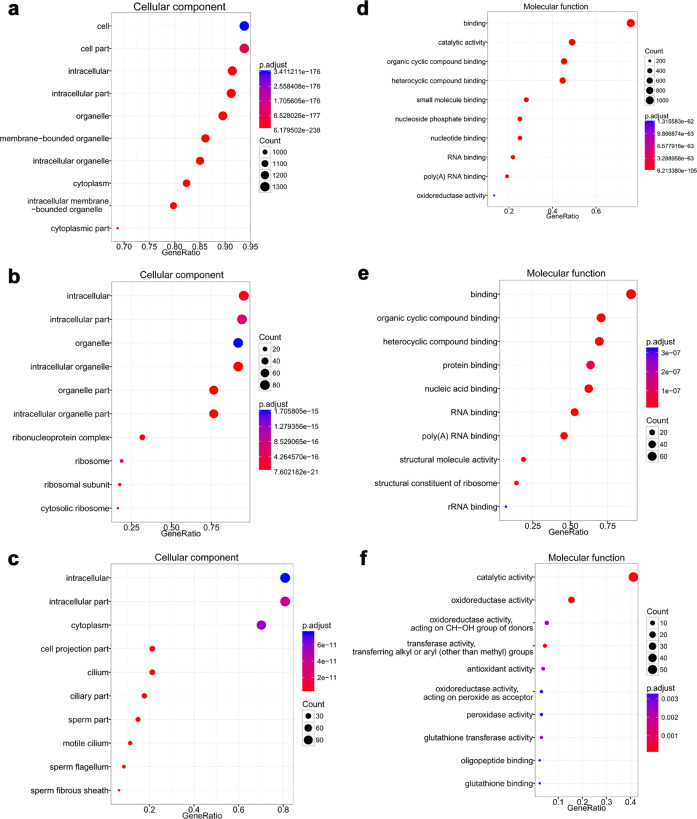
Cellular components and molecular functions of GO analysis. **a**–**c** Cellular components of total identified proteins, proteins upregulated, and proteins downregulated upon H_2_O_2_ treatment. **d**–**f** Molecular functions of total identified proteins, proteins upregulated, and proteins downregulated upon H_2_O_2_ treatment.

Both the total identified proteins and the differentially expressed proteins were mainly enriched for the GO terms associated with cell part, intracellular part, and organelle. The cytoplasmic compartment was the most represented by the identified proteins (78.21%, 1145/1464), followed by the membrane (60.45%, 885/1464) and nuclear (22.81%, 334/1464) compartments. H_2_O_2_ treatment increased the proportion of nuclear proteins (45.42%, 109/240; *P* < 0.001) and decreased the proportion of cytoplasmic proteins (68.33%, 164/240; *P* = 0.001). The increased expression of Ranbp2, Lmna, Ipo5, and Lrrc59, which play positive roles in regulating protein import into the nucleus, suggested that oxidative stress triggered nuclear-cytoplasmic transport participating in DNA/chromosome repair in IVF-derived embryos^24,25^. Furthermore, the upregulated proteins had a special relationship with the ribonucleoprotein complex (ribosome), whereas the downregulated proteins were more related to the cilium (Fig. 6a–c). H_2_O_2_ treatment principally caused significant increases in the expression of ribosomal proteins, including small (Rps3a1, Rps7, Rps11, Rps14, Rps18, and Rps20) and large (Rpl6, Rpl8, Rpl10a, Rpl11, Rpl15, Rpl23, Rpl27, Rpl34, Rpl36a, and Rpl37a) ribosomal subunits and heterogeneous nuclear ribonucleoproteins (hnRNP A3, hnRNP F, hnRNP K, and hnRNP U).

#### Molecular function

Through analysis of molecular functions, we found that most of the total identified proteins were enriched for the binding function term (70.8%, 1037/1464), followed by catalytic activity (45.7%, 669/1464). A total of 82.8% (77/93) of the upregulated proteins were assigned to a binding function; in particular, H_2_O_2_ treatment increased the expression of proteins acting as structural constituents of the ribosome and involved in rRNA binding. In addition, 61.2% (90/147) of the downregulated proteins were assigned to catalytic activity; in particular, H_2_O_2_ treatment decreased the expression of proteins involved in antioxidant activity, mainly glutathione peroxidase activity and glutathione transferase activity, resulting in the accumulation of ROS in IVF-derived embryos (Fig. 6d–f).

#### Biological process

**Fig. 7:**
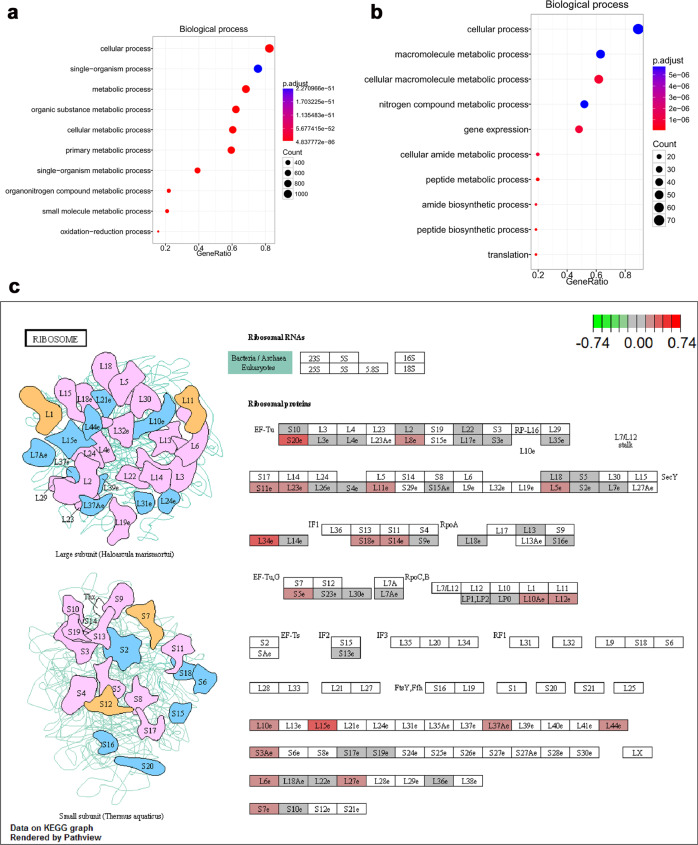
Biological process of GO analysis and KEGG analysis. **a**, **b** Biological processes of total identified proteins and proteins upregulated upon H_2_O_2_ treatment. **c** KEGG pathway map. KEGG analysis showed that the upregulated proteins were mainly involved in ribosome biogenesis.

The GO biological process analysis indicated that the top three biological process terms of all identified proteins were cell process, single-organism process, and metabolic process (Fig. 7a). While the downregulated proteins generally participated in the biological process of production^7^, the 93 upregulated proteins were largely associated with gene expression and macromolecule metabolic processes such as DNA repair and replication and protein synthesis and degradation (Fig. 7b).

To better understand the involved pathways, these differentially expressed proteins were further analyzed by KEGG pathway enrichment analysis. According to the KEGG pathway maps, the major upregulated proteins were annotated into the ribosome pathway (Fig. 7c), while the downregulated proteins were abundant in the glutathione metabolism pathway (Fig. 1a). Thus, the GO and KEGG functional enrichment analyses consistently revealed that suppression of glutathione peroxidase and glutathione transferase activity was responsible for the overproduction of ROS and subsequent responses to oxidative stress, which plays critical roles in the promotion of ribosomal protein expression (ribosome biogenesis) and ribosome function in IVF-derived embryos.

The ribosome is a molecular machine in charge of protein synthesis^10^. It can be seen from the above functional analysis that ribosomal proteins were upregulated by ROS, which may have enhanced ribosome function to enable synthesis of an abundance of repair proteins to correct the damaged DNA/chromosomes in IVF-derived embryos. Three main molecular mechanisms were implicated in the repair of oxidative DNA damage in IVF-derived embryos. First, the upregulation of Psme3, hnRNP K, and H2BC3 suggests that the ATM/P53-dependent DNA damage checkpoint is fully activated and that G_2_/M cell cycle arrest is maintained for several hours until repair is effected and cells reenter the cell cycle^26^. Our previous results confirmed that ROS caused G_2_/M cell cycle arrest via the ATM-Chk1-Cdc25-Cdc2 pathway, providing more time for DNA repair in IVF mouse zygotes^6^. Second, the increase in Prkar2b expression and the corresponding decrease in Akap4 expression lead to dysfunction of cAMP-dependent PKA signaling, which engages in DNA repair by phosphorylating DNA damage response elements, such as ATR/ATM^27^. Third, the elevated expression of Ranbp2, Dek, and H2BC3 promotes DNA double-strand break repair via nonhomologous end-joining, but not homologous recombination, during G_2_/M cell cycle arrest^28^.

### Possible mechanisms involved in tumorigenesis in offspring derived from oxidatively damaged IVF embryos

From the abovementioned 240 differentially expressed proteins, we enriched 58 tumor-related proteins, including 45 upregulated and 13 downregulated proteins, and analyzed tumor-related signaling pathways (Supplementary Tables 3 and 4). In addition to oncogene activation and tumor suppressor gene inactivation, several other possible signaling pathways involved in tumorigenesis in offspring derived from oxidatively damaged embryos were identified. The first identified pathway was the ribosome signaling pathway. The upregulation of ribosomal proteins (large 60S subunit, small 40S subunit, and hnRNPs) suggested that oxidative damage stimulated ribosome biogenesis in IVF-derived embryos, and persistent hyperactivation of ribosome biogenesis conferred many competitive advantages to cancer cells^12^. Moreover, Nucleolin, n-Myc, Ddx17, and Syne2 participate in cancer progression when overexpressed by altering ribosome biogenesis^29,30^. The second potential pathway identified was the Wnt signaling pathway. The upregulated expression of Nucleolin, Prkcsh, Prkar2b, and Zbed3 and the downregulated expression of Nfatc4 promoted cancer cell proliferation, migration, and invasion by activating the Wnt/β-Catenin signaling pathway^31,32^. The third identified pathway was the TGF-β1 signaling pathway. Oxidative stress results in increased expression of calreticulin, Psme3, and vimentin, which regulate TGF-β1-induced proliferation and epithelial-mesenchymal transition (EMT) by modulating Smad signaling^33,34^. Besides, Srsf5, Rdx, Tra2b, and NuMA1 function as novel oncogenes and are upregulated by ROS to regulate the cell cycle and apoptosis in IVF-derived embryos^35,36^.

We applied MetaCore software to analyze the protein–protein interaction (PPI) networks of the differentially expressed proteins and identified a total of 59 biological networks. Within the networks analyzed, 23 pathways were associated with cellular metabolic processes, especially the cellular macromolecule biosynthetic process, which produces DNA repair products but also provides sufficient protein for tumor cells to grow and proliferate (Supplementary Fig. 1). Ten pathways were associated with the cellular response to oxidative stress. Ten tumor-related pathways were primarily enriched for the regulation of cell apoptotic process (4 pathways), cell proliferation (2), cell cycle (1), cell differentiation (1), mesenchyme migration (1), and other terms, such as DNA conformation change (1), suggesting that the ribosome, Wnt, and TGF-β1 signaling pathways may promote tumorigenesis by regulating apoptosis (Supplementary Figs. 2 and 3).

### mRNA levels of the key ribosome-/tumor-related differentially expressed proteins Nucleolin, n-Myc, Rpl15, Rpl36a, hnRNP K, β-Catenin, and TGF-β1

**Fig. 8:**
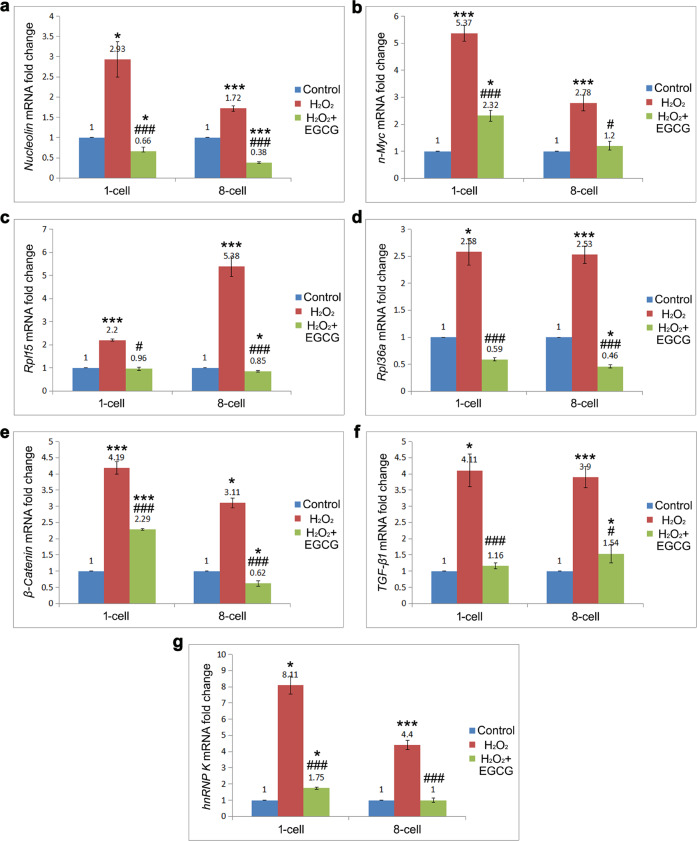
mRNA levels of the key ribosome-/tumor-related differentially expressed proteins Nucleolin, n-Myc, Rpl15, Rpl36a, hnRNP K, β-Catenin, and TGF-β1 by RT–qPCR. Data are expressed as the means ± SDs, collected from three independent experiments. Differences among groups were analyzed using Student’s *t* test. *P < 0.05 and ***P < 0.001 vs. control, ^#^*P* < 0.05 and ^###^*P* < 0.001 vs. H_2_O_2_ treatment.

As we have noted previously, Nucleolin and n-Myc play positive roles in regulating the expression of nearly all ribosomal proteins, including Rpl15, Rpl36a, and hnRNP K, which may participate in tumorigenesis in offspring derived from oxidatively damaged embryos by cross-interacting with the Wnt and TGF-β1 pathways. Therefore, RT–qPCR was used to determine the mRNA expression levels of these key tumor-related differentially expressed proteins in 1-cell (M phase) and 8-cell embryos from different groups (Fig. 8a–g). The results showed high mRNA expression of *Nucleolin*, *n-Myc*, *Rpl15*, *Rpl36a*, *hnRNP K*, *β-Catenin*, and *TGF-β1* in the 1-cell and 8-cell embryos of the H_2_O_2_-treated group compared with the control group (*P* < 0.05), which was consistent with the iTRAQ findings.

However, EGCG, due to its strong antioxidant activity, prevented the oxidative damage-induced dysfunction of ribosome biogenesis. The mRNA expression of *Nucleolin*, *n-Myc*, *Rpl15*, *Rpl36a*, *hnRNP K*, *β-Catenin*, and *TGF-β1* was significantly lower in the 1-cell and 8-cell embryos of the H_2_O_2_/EGCG-treated group than in those of the H_2_O_2_-treated group (*P* < 0.05), suggesting that EGCG treatment regulated the abnormal expression of ribosome-/tumor-related genes in oxidatively damaged IVF embryos. Furthermore, the levels of most ribosome-/tumor-related genes tended to decrease with the development of oxidatively damaged embryos but were still significantly higher than those in the control group (*P* < 0.05). Only *Rpl15* expression increased remarkably from the 1-cell to 8-cell stages, suggesting that Rpl15 may be the most critical ribosomal protein in carcinogenesis in IVF-derived offspring. The *Rpl15* level was restored to the normal level by EGCG supplementation under oxidative stress.

## Discussion

A growing number of scientists are beginning to realize that IVF-conceived children are at an increased risk of pediatric neoplasms^1–4^. To date, research has focused mostly on the prevalence of neoplasms and the factors that induce them (e.g., causes of infertility, use of gonadotropins for controlled ovarian stimulation, and application of IVF or intracytoplasmic sperm injection). There is a lack of animal experimental research to reveal the molecular and genetic mechanisms of tumorigenesis in IVF-derived offspring. In this study, the in vitro culture conditions led to excessive production of ROS, which caused DNA damage and aneuploidy formation in IVF-derived embryos. The association of DNA damage/chromosome aneuploidy and tumorigenesis has long been recognized^8,9^. Oxidative damage may be one of the most important reasons for tumorigenesis in IVF-conceived children. Therefore, we used this model of oxidatively damaged IVF mouse embryos to study the tumorigenic mechanisms. It is also the first time that animal experiments have been used to elucidate the possible pathogenesis of tumors in offspring derived from oxidatively damaged IVF embryos. We concluded that ROS-induced damage to DNA and chromosomes promotes tumor progression mainly via alterations in ribosome biogenesis as well as via activation of the Wnt/β-Catenin and TGF-β1/Smad signaling pathways.

Ribosomes are RNA-protein complexes responsible for protein synthesis^10^. This study found that both rRNAs and ribosomal proteins can be chemically modified by ROS, which prompts ribosomes to synthesize an abundance of DNA repair proteins to correct damaged DNA in IVF-derived embryos at an early developmental stage. However, the increased percentages of γH2AX- and TUNEL-positive cells in the blastocyst suggested that DNA repair was insufficient, possibly resulting in persistent hyperactivation of ribosome biogenesis. Increasing evidence shows that persistent hyperactive ribosome biogenesis confers competitive advantages to cancer cells^37^. In one study, RNA sequencing of freshly isolated circulating breast cancer cells revealed a subset with strong ribosome and protein synthesis signatures that were correlated with poor clinical outcomes^38^. The essential role of increased ribosome biogenesis and protein synthesis in sustaining tumor cell growth and proliferation is well established^39^. In addition, the execution of EMT, a migratory cellular program associated with tumor development and metastasis, is fueled by upregulation of ribosomal proteins^40^. We also found that several ribosome-/tumor-related genes were particularly actively expressed even in 8-cell preimplantation embryos, and persistent hyperactivation of ribosome biogenesis could play essential roles in the initiation and progression of cancers in IVF-derived offspring.

Rpl15 was identified as the most significantly upregulated ribosomal protein in our iTRAQ experiment, and RT–qPCR analysis revealed stepwise upregulation of *Rpl15* from the 1-cell to 8-cell stages in response to oxidative damage. It has been found that Rpl15 can promote metastatic growth in multiple organs^38,41^. The number of nucleoli and the expression of nucleolar proteins increased when Rpl15 was overexpressed, and abnormal increases in nucleolar size and number caused by dysregulation of ribosome biogenesis have emerged as hallmarks of the majority of spontaneous cancers^42^. Mechanistically, overexpression of Rpl15 selectively enhances translation of other ribosomal proteins^38^. Furthermore, *Rpl15* siRNA-mediated downregulation induces cell cycle arrest by inhibiting cyclin-dependent kinases and results in a significant increase in the percentage of apoptosis in cancer cells^38,41^. Therefore, Rpl15 is the most critical ribosomal protein in carcinogenesis, increasing the risk of tumor formation in IVF-derived offspring.

The mechanisms of hyperactive ribosome biogenesis in response to ROS in IVF-derived embryos are associated with several features. ROS upregulate the expression of Nucleolin, n-Myc, Ddx17, and Syne2, which are most widely known for their positive participation in steps throughout ribosome biogenesis, including synthesis and processing of rRNAs, assembly of ribosomal proteins, transport to the cytoplasm and association of ribosomal subunits^29,30,43,44^. On the other hand, the Wnt/β-Catenin/c-Myc signaling pathway is the major pathway that works in concert with each of the three RNA polymerases (RNA Pol I, II, and III) to regulate ribosome biogenesis^45^. Decreases in ribosomal proteins are accompanied by decreased expression and activity of β-Catenin^46^. In our oxidatively damaged IVF embryos, the upregulation of Nucleolin promoted Wnt/β-Catenin signaling, which globally affects multiple steps in ribosome biogenesis and enhances the expression of nearly all ribosomal proteins via a Myc-dependent pathway^47^. Myc (both c-Myc and n-Myc) has been shown to serve as a direct positive regulator of ribosome biogenesis^30^. ROS caused an increase in n-Myc (not c-Myc), suggesting that n-Myc may be the critical molecule by which Wnt/β-Catenin signaling regulates ribosome biogenesis in preimplantation embryos.

Ribosome biogenesis in cancer depends on multiple factors. In addition to protein synthesis function, most ribosomal proteins can influence cellular processes by performing extraribosomal regulatory functions involving binding to select critical target mRNAs^14^. For example, hyperactive ribosome biogenesis can drive activation of oncogenes (e.g., *En2* and *n-Myc*) and silencing of tumor suppressor genes (e.g., *Per2* and *Diras2*) in oxidatively damaged IVF embryos^48,49^. More importantly, hyperactive ribosome biogenesis can trigger the concomitant activation of tumor-related signaling pathways. Here, we show, for the first time, that oxidative damage establishes oncogenic cooperation among the ribosome, Wnt, and TGF-β1 signaling pathways in IVF-derived embryos. As mentioned above, oxidative damage activates the Wnt/β-Catenin signaling pathway, which stimulates the expression of ribosomal proteins^47^. Conversely, knockdown of ribosomal proteins downregulates β-Catenin expression and blocks Wnt signaling^50^. In addition, ribosomal proteins, which are frequently amplified in many types of human cancers, mediate the TGF-β1/Smad signaling pathway. In line with our results, Rpl22l1 binds to intronic sequences of *Smad2* premRNA and modulates splicing of the premRNA encoding *Smad2*, an essential transcriptional effector of TGF-β signaling; Smad2 phosphorylation and expression are reduced upon knockdown of Rpl22l1^51^. In contrast, EMT induction by TGF-β1 in cancer cells primarily mediates translational downregulation of ribosomal proteins^38^. Consequently, the persistent hyperactivation of ribosome biogenesis plays a tumor-promoting role in offspring derived from oxidatively damaged IVF embryos by cross-interacting with the Wnt/β-Catenin and TGF-β1/Smad pathways.

Heterogeneous nuclear ribonucleoprotein K (hnRNP K) is a DNA/RNA-binding protein that regulates a wide range of biological processes and disease pathogeneses. Many studies have identified *hnRNP K* as an oncogene, as it is overexpressed in cancer tissues compared with nonneoplastic tissues, and its expression level is related to the prognoses of different types of malignancies^52^. hnRNP K is a novel internal ribosomal entry site trans-acting factor^53^. hnRNP K is also central to regulating activation of the Wnt/β-Catenin and TGF-β1/Smad signaling pathways^54–57^. β-Catenin, which accumulates in the nucleus, activates Wnt signaling through complexation with hnRNP K^54,55^. hnRNP K promotes the TGF-β1-induced EMT process in lung cancer cells; the EMT phenotype of lung cancer cells can be increased via self-production of TGF-β1 and significantly decreased by silencing hnRNP K expression^56,57^. Therefore, through cross-talk among the β-Catenin, ribosome, and TGF-β1 pathways, hnRNP K may be involved in processes critical to ribosome biogenesis and cancer progression in oxidatively damaged IVF embryos.

Due to its excellent antioxidative and antitumor properties, efforts have been made to use EGCG to produce healthy embryos and offspring. In vitro culture conditions stress mouse embryos and contribute to the reduced cell numbers of blastocysts. Blastocyst biopsy (in which a small number of cells are taken from the trophectoderm at the blastocyst stage) may not add additional risk of poor neonatal outcomes^58^. Nevertheless, the blastocyst total cell number and the ICM-trophectoderm score are correlated with positive outcomes of clinical pregnancy rate and live birth rate^59^. Mouse experiments have also revealed that offspring derived from blastocysts with fewer cells display decreased spleen weights, decreased values of several organ-to-body weight ratios (for the heart, lungs, spleen and liver), reduced crown-rump lengths, abnormal limb development and other features^60^. However, there is no literature on the threshold number of blastocyst cells necessary to prevent negative impacts on clinical outcomes and offspring health. In this research, we treated mouse zygotes with a low concentration (0.03 mM) of H_2_O_2_ to create a model strongly recapitulating the clinically observed phenomenon of oxidative damage in IVF embryos and found that the ROS level in the H_2_O_2_/EGCG-treated group was close to that in the EGCG-treated group and that the total cell numbers of IVF blastocysts in both groups were nearly 1.5-fold higher than that in the control group. Thus, EGCG has a trophic pro-proliferative effect, increasing the blastocyst cell number and improving the clinical outcomes of IVF.

In addition, we discovered that in vitro culture conditions contribute to the reduced cell number of blastocysts as well as to the increased numbers of γH2AX-positive cells and TUNEL-positive cells in blastocysts. These results indicate that preimplantation blastocysts may consist of oxidatively damaged and apoptotic cells that are harmful for IVF clinical outcomes. However, after EGCG treatment, embryo developmental competence was improved, as indicated by the increased ICM and total cell number, in association with the reductions in the numbers of damaged and apoptotic cells in blastocysts developed from H_2_O_2_/EGCG-treated zygotes. Furthermore, EGCG supplementation reversed the effects of ROS on the expression of ribosome-/tumor-related proteins in preimplantation embryos. Our data show, for the first time, that EGCG is beneficial for protecting IVF embryos from ROS-induced DNA/chromosome damage and maintaining ribosome biosynthesis at a level appropriate for the growth of embryonic cells, representing a new strategy for preventing offspring tumorigenesis.

In conclusion, transient increases in ribosomal protein synthesis are beneficial for DNA repair. However, as a consequence of insufficient or inefficient DNA repair, persistent hyperactivation of ribosome biogenesis is one of the most important mechanisms of tumorigenesis in offspring derived from oxidatively damaged IVF embryos. Rpl15 plays a critical role in accelerating tumor progression by selectively enhancing the translation of other ribosomal proteins and cell cycle/apoptosis regulators. Oxidative damage establishes oncogenic cooperation among the β-Catenin, ribosome, and TGF-β1 signaling pathways. hnRNP K may be essential for cross-talk among the Wnt/β-Catenin, ribosome, and TGF-β1/Smad pathways. This study advances our current understanding of the molecular mechanisms of tumorigenesis in IVF-derived offspring. More importantly, our findings that EGCG increased the blastocyst formation rate/blastocyst undamaged cell number and inhibited hyperactivation of ribosome biogenesis provide a scientific basis for the use of EGCG to optimize the in vitro culture conditions of human embryos. Frankly speaking, the results of this paper were obtained from animal experimental models, which is the greatest limitation of this study. Given ethical factors and the scarcity of human samples, we chose not to conduct experiments directly on human embryos. To clarify whether similar results occur in human embryos, we will use discarded IVF embryos to observe the effects of oxidative damage on ribosome biogenesis and the carcinogenic effects of the interaction of hyperactive ribosome biogenesis and abnormal X chromosome inactivation.

## Supplementary information

SUPPLEMENTAL MATERIAL
