# Supplementary material for: Oxidative damage-induced hyperactive ribosome biogenesis participates in tumorigenesis of offspring by cross-interacting with the Wnt and TGF-β1 pathways in IVF embryos
Source: Exp Mol Med. 2021 Nov 30;53(11):1792–806. doi: 10.1038/s12276-021-00700-0 (PMC8640061; doi:10.1038/s12276-021-00700-0)
Supplement: Supplementary file 1 — SUPPLEMENTAL MATERIAL [file 12276_2021_700_MOESM1_ESM.pdf]

Supplementary Tables

Supplementary Table 1 The main reagents used in this paper

Reagents	Supplier
HEPES buffered Human tubal fluid (HTF)	Cooper Surgical (USA)
Pregnant mare serum gonadotropin (PMSG)	Ningbo Second Hormone Factory (China)
Human chorionic gonadotropin (HCG)	Ningbo Second Hormone Factory (China)
Epigallocatechin gallate (EGCG)	Sigma-Aldrich E4143 (USA)
DCFH-DA	Sigma-Aldrich D6883 (USA)
Rabbit monoclonal antibody against OTC-4	Abcam Cat# ab181557 (UK)
Rabbit polyclonal antibody against gamma H2A.X	Abcam Cat# ab11174 (UK)
Goat polyclonal secondary antibody against rabbit IgG (Alexa Fluor® 488)	Abcam Cat# ab150077 (UK)
Mouse monoclonal antibody against α-tublin (FITC-conjugated)	Abcam Cat# ab64503 (UK)
Rabbit polyclonal antibody against MAT2	Abcam Cat# ab186129 (UK)
Rabbit polyclonal antibody against GAPDH	Abcam Cat# ab9485 (UK)
Goat Anti-Rabbit IgG (HRP)	Abcam Cat# ab7090 (UK)
In situ cell death detection kit (fluorescein)	Roche 11684795910 (China)
iTRAQ reagents	Applied Biosystems Incorporation (USA)
RIPA lysis buffffer	Beyotime (China)
Protease and phosphatase inhibitor cocktail	Beyotime (China)
RNAprep pure Micro Kit	Tiangen Biotech DP420 (China)
FastKing gDNA Dispelling RT SuperMix	Tiangen Biotech KR118 (China)
Talent qPCR PreMix (SYBR Green)	Tiangen Biotech FP209 (China)

Supplementary Table 2 The primer sequences

	Forward primer (5’-3’)	Reverse primer (5’-3’)
**Nucleolin**	AAAGGCAAAAAGGCTACCACA	GGAATGACTTTGGCTGGTGTAA
**β-Catenin**	ACGGCCAGGTCATCACTATTG	CAAGAAGGAAGGCTGGAAAAGA
**n-Myc**	CCTCACTCCTAATCCGGTCAT	GTGCTGTAGTTTTTCGTTCACTG
**Rpl15**	GTCCGCTGTTGGCAATACC	ACATAGCCTTGCTTAGCCTTGTA
**Rpl36a**	GGAAAGCGGCGTTACGACA	TCGCCTCCCAATTCAAAATGC
**hnRNP K**	GGAGGGGTTCGTGGTGAAG	GAACACCTGATGTGCCATTTTG
**TGF-β1**	CTTCAATACGTCAGACATTCGGG	GTAACGCCAGGAATTGTTGCTA
**GAPDH**	AGCAGTCCCGTACACTGGCAAAC	TCTGTGGTGATGTAAATGTCCTCT
**β-acting**	TGCTGTCCCTGTATGCCTCT	TTGATGTCACGCACGATTTC

Supplementary Table 3 The upregulation of ribosome biogenesis-related proteins

	Proteins	T vs. C
Large 60S subunit	Rpl6	1.556
	Rpl8	1.534
	Rpl10a	1.951
	Rpl11	1.971
	Rpl15	2.094
	Rpl23	1.541
	Rpl27	1.706
	Rpl34	1.988
	Rpl36a	1.819
	Rpl37a	1.711
Small 40S subunit	Rps3a1	1.571
	Rps7	1.698
	Rps11	1.664
	Rps14	1.67
	Rps18	1.541
	Rps20	2.058
Heterogeneous nuclear ribonucleoprotein	hnRNP A3	1.74
	hnRNP F	1.514
	hnRNP K	1.913
	hnRNP U	1.711
Ribosome biogenesis-related proteins	Nucleolin	1.541
	Syne2	1.609
	Ddx17	1.668
	Ddx39	1.689
	Rrbp1	1.699
	n-Myc	1.732

Supplementary Table 4 The dysregulation of tumor-related proteins

		Proteins	T vs. C
The upregulation of tumor-related proteins	Wnt/β-Catenin signaling pathway-related proteins	Nucleolin	1.541
		Prkcsh	1.577
		Prkar2b	1.519
		Zbed3	2.038
	TGF-β1/Smad signaling pathway-related proteins	Calreticulin	3.603
		Psme3	1.905
		Vimentin	1.504
	Cell cycle and apoptosis-related proteins	Srsf5	1.957
		Rdx	1.72
		Tra2b	2.064
		NuMA1	1.645
	Src family kinases	Fam120a	1.917
		Nt5dc2	1.55
	Oncogenes	En2	5.49
		Jak3	2.649
		Ipo5	1.564
		Dek	1.568
		Raly	1.521
		Ranbp2	1.728
		Hsp90b1	1.628
The downregulation of tumor-related proteins	Tumor suppressor genes	Diras2	0.579
		Nfatc4[1]	0.602
		Cpt1b	0.641
		Mlf1	0.589
		Per2	0.572
		Pgrmc1	0.61
		Prss21	0.536
		Asrgl1	0.651
		Pacrg	0.53
		Csl	0.549
		Tepp	0.645
		Ptchd3	0.629
		P3h2	0.417

[1] The downregulation of Nfatc4 also promotes the Wnt/β-Catenin signaling pathway.

Supplementary Figures

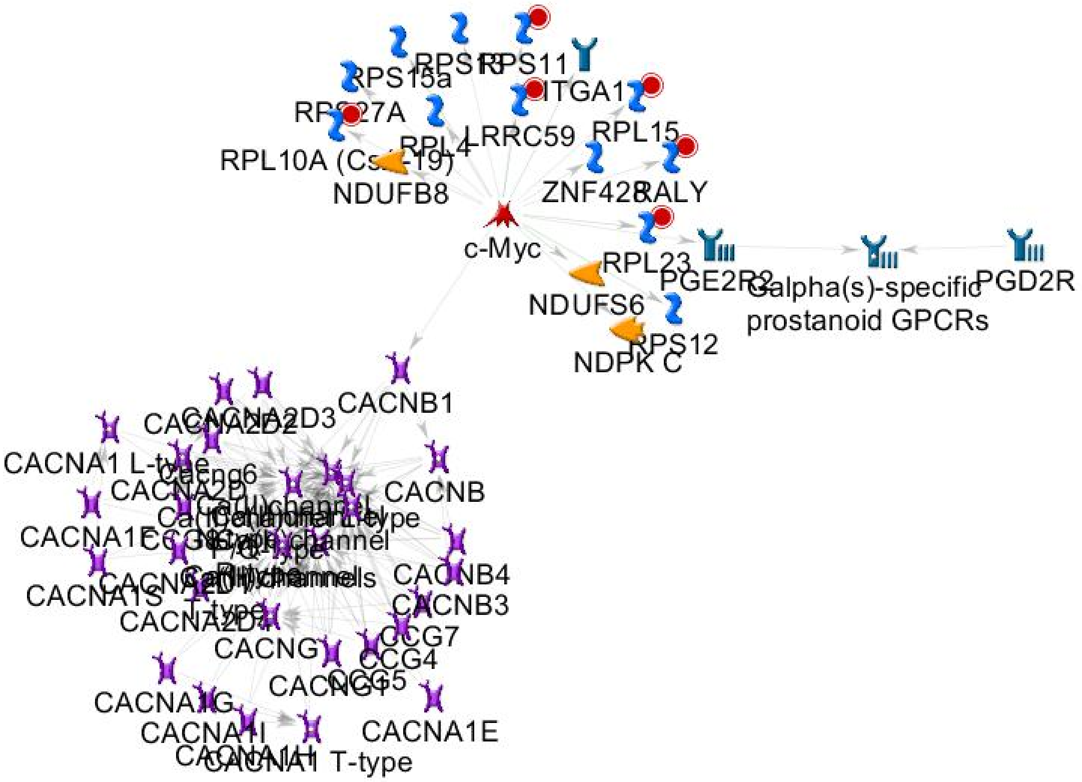

Supplementary Fig. 1 The biological network of the key differentially expressed proteins Rps11, Lrrc59, Rpl15, Raly, and Rpl10a was mainly enriched in the positive regulation of macromolecule biosynthetic proces. Thick cyan lines indicate the fragments of canonical pathways. Upregulated genes are marked with red circles, while downregulated one are with blue circles.

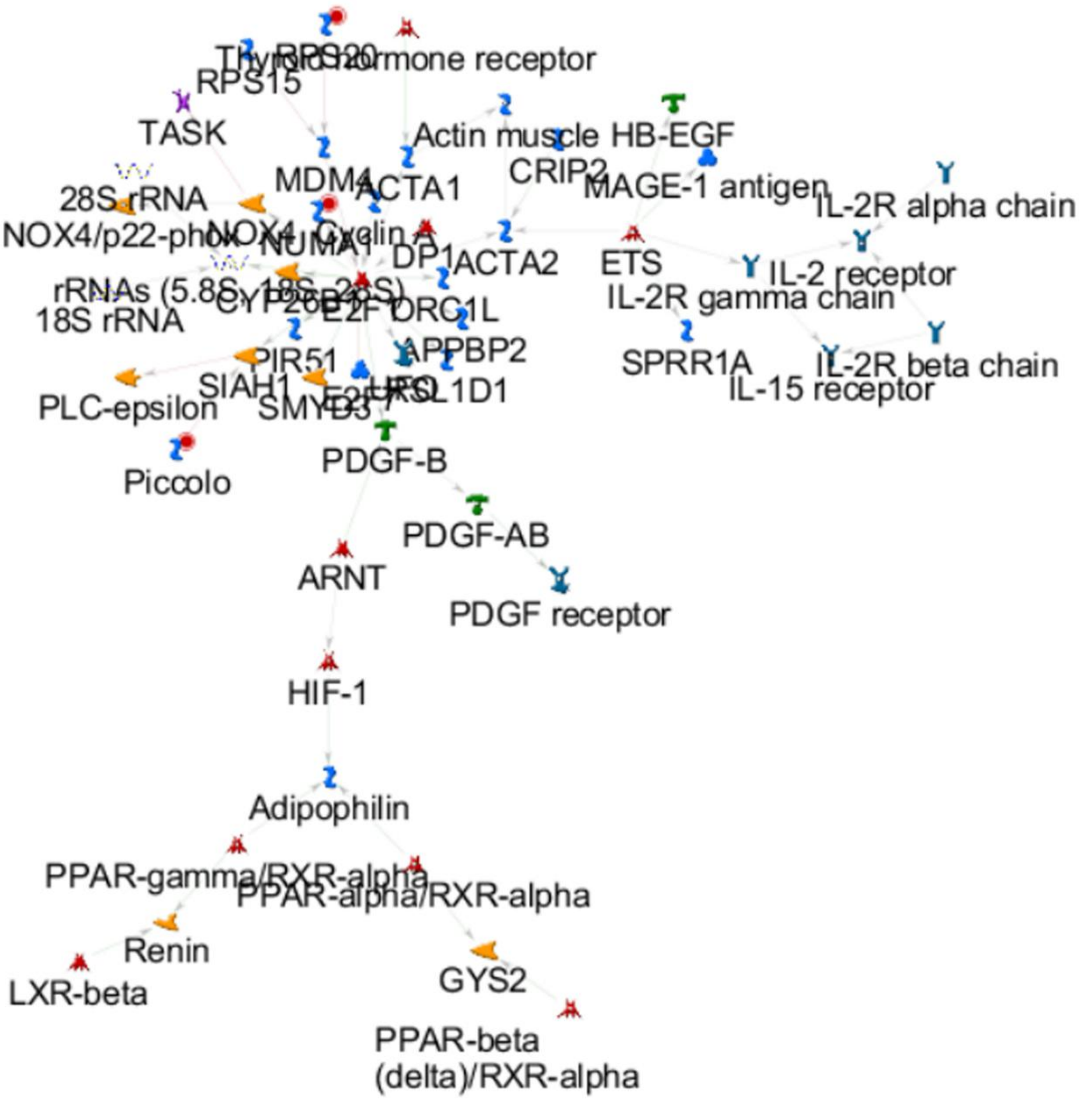

Supplementary Fig. 2 The biological network of the key differentially expressed proteins Rps20, NuMA1, Piccolo, E2f1, and Ets was mainly enriched in the regulation of cell proliferation. Thick cyan lines indicate the fragments of canonical pathways. Upregulated genes are marked with red circles, while downregulated one are with blue circles.

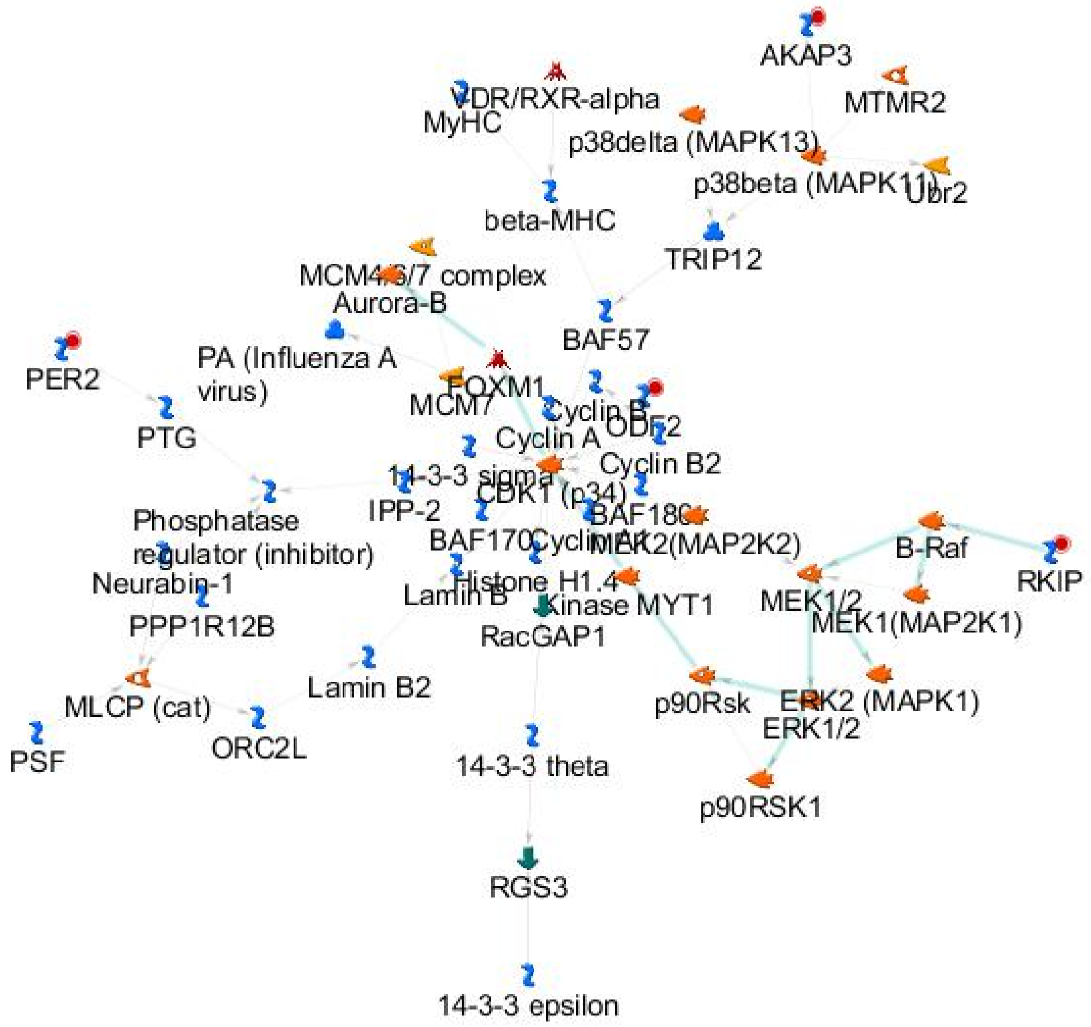

Supplementary Fig. 3 The biological network of the key differentially expressed proteins Odf2, Akap3, Per2, Rkip, and Cdk1 (p34) was mainly enriched in the regulation of cell cycle. Thick cyan lines indicate the fragments of canonical pathways. Upregulated genes are marked with red circles, while downregulated one are with blue circles.
